# Upregulation of NR2A in Glutamatergic VTA Neurons Contributes to Chronic Visceral Pain in Male Mice

**DOI:** 10.1007/s12264-025-01402-7

**Published:** 2025-04-28

**Authors:** Meng-Ge Li, Shu-Ting Qu, Yang Yu, Zhenhua Xu, Fu-Chao Zhang, Yong-Chang Li, Rong Gao, Guang-Yin Xu

**Affiliations:** 1 Center of Translational Medicine, The Zhangjiagang Affiliated Hospital of Soochow University, Zhangjiagang, 215600 China; 2 Jiangsu Key Laboratory of Neuropsychiatric Diseases and Institute of Neuroscience, Soochow University, Suzhou, 215123 China; 3 Department of Gastroenterology, Suzhou Dushu Lake Hospital, The Forth Affiliated Hospital of Soochow University, Suzhou, 215123 China

**Keywords:** Irritable bowel syndrome, Chronic visceral pain, Ventral tegmental area, Glutamatergic neurons, NMDA receptor 2A subunit

## Abstract

Chronic visceral pain is a persistent and debilitating condition arising from dysfunction or sensitization of the visceral organs and their associated nervous pathways. Increasing evidence suggests that imbalances in central nervous system function play an essential role in the progression of visceral pain, but the exact mechanisms underlying the neural circuitry and molecular targets remain largely unexplored. In the present study, the ventral tegmental area (VTA) was shown to mediate visceral pain in mice. Visceral pain stimulation increased c-Fos expression and Ca^2+^ activity of glutamatergic VTA neurons, and optogenetic modulation of glutamatergic VTA neurons altered visceral pain. In particular, the upregulation of NMDA receptor 2A (NR2A) subunits within the VTA resulted in visceral pain in mice. Administration of a selective NR2A inhibitor decreased the number of visceral pain-induced c-Fos positive neurons and attenuated visceral pain. Pharmacology combined with chemogenetics further demonstrated that glutamatergic VTA neurons regulated visceral pain behaviors based on NR2A. In summary, our findings demonstrated that the upregulation of NR2A in glutamatergic VTA neurons plays a critical role in visceral pain. These insights provide a foundation for further comprehension of the neural circuits and molecular targets involved in chronic visceral pain and may pave the way for targeted therapies in chronic visceral pain.

**Supplementary Information:**

The online version contains supplementary material available at 10.1007/s12264-025-01402-7.

## Introduction

Irritable bowel syndrome (IBS) is a functional gastrointestinal disorder with a global prevalence rate of 10%–25% [1, 2]. Chronic visceral pain is one of the most common reasons for which IBS patients seek medical assistance. It is characterized by poor localization, referral to distant sites, and frequent involvement of autonomic and emotional components, making its diagnosis and treatment particularly challenging. The pathophysiology of chronic visceral pain is multifaceted, involving the peripheral sensitization of visceral afferents, central sensitization in the spinal cord, and dysregulation of the brain-gut axis [3]. Despite significant advances in understanding the mechanisms underlying chronic visceral pain, effective treatment options remain limited. Moreover, current medications for managing chronic visceral pain in IBS patients are often ineffective and associated with significant side-effects [4–7]. Therefore, further research into the molecular and cellular mechanisms of visceral pain is essential to develop novel therapeutic strategies and improve patient outcomes.

Pain involves a complex integration of nociception, consciousness, and emotion, mediated by intricate brain networks [8]. When pain signals originate from the viscera, they can trigger both peripheral and central sensitization, leading to heightened sensitivity in specific neural circuits. This process may ultimately result in chronic visceral hypersensitivity [9], a hallmark clinical feature commonly reported in IBS patients [10]. Studies have shown that the dopamine system in the ventral tegmental area (VTA) plays a role in regulating paraventricular nucleus corticotropin-releasing hormone in a rat model with chronic visceral pain [11]. In the mammalian brain, several regions have been implicated in visceral pain processing, including the paraventricular hypothalamic nucleus, lateral septal ventral nucleus, anterior cingulate cortex, and claustrum [12, 13]. While classical studies on visceral pain have elucidated how the central nervous system modulates pain transmission through structural projections between nuclei, the underlying therapeutic mechanisms remain poorly understood.

The VTA is a heterogeneous region composed of glutamatergic neurons, gamma-aminobutyric acid (GABAergic) neurons, and dopaminergic neurons [14, 15]. Historically, studies have primarily focused on the dopaminergic and GABAergic neurons within the VTA [16–19]. However, increasing evidence underscores the critical role of glutamatergic VTA neurons in regulating diverse behaviors, including pain perception, reward aversion, defense mechanisms, sleep, and arousal [20–22]. Notably, a recent study confirmed the involvement of glutamatergic VTA neurons in pain regulation in mice with chronic constriction injury [23]. Despite this progress, the potential role of these neurons in chronic visceral pain regulation remains largely unexplored. A key mechanism underlying the activation of glutamatergic neurons is the binding of glutamate to its receptors [24, 25]. These receptors are classified into metabotropic glutamate receptors and ionotropic glutamate receptors (iGluRs) based on their mechanisms of action [26]. Understanding the specific roles of these receptors in the activity of glutamatergic VTA neurons could provide valuable insights into their function in chronic pain modulation.

N-methyl-D-aspartic acid receptors (NMDARs) are a subtype of iGluRs, which include NR1, NR2A, NR2B, NR2C, NR2D, NR3A, and NR3B subunits [27]. The typical NMDAR is a tetramer composed of two NR1 subunits and two NR2 subunits (2A-2D), with random combinations of NR2 subunits [28, 29]. These receptors are widely expressed in the central nervous system and play an important role in excitatory synaptic transmission [30]. AMPA (α-amino-3-hydroxyl-5-methyl-4-isoxazole-propionate) receptors are another subtype of iGluRs that mediate fast excitatory synaptic transmission in the central nervous system (CNS), and are involved in excitotoxicity mediated by glutamatergic neurotransmission [24]. Glutamate binding to NMDARs triggers the opening of Ca^2+^ channels, leading to increased CNS excitability [31]. Studies have shown that NMDAR activation can induce and maintain central sensitization, and the upregulation of NMDARs contributes to visceral hypersensitivity [28, 32, 33]. However, whether NMDARs in the VTA play a regulatory role in chronic visceral pain remains unknown.

In this study, we provide compelling evidence that inhibition of NR2A reduces VTA activation and alleviates chronic visceral pain. Our findings suggest that the upregulation of NR2A in glutamatergic VTA neurons plays a critical role in the modulation of visceral pain. These insights contribute to the understanding of the central mechanisms underlying visceral pain in IBS patients and offer potential therapeutic targets for the development of effective treatments.

## Materials and Methods

### Animals

The experimental animal strain was male SPF C57BL/6J mice aged 6–12 weeks. The mice were raised in strict accordance with the feeding standard, and the density in each cage was no more than 5 animals. All mice were housed under a stable temperature of 24 °C ± 1 °C and constant humidity between 40% and 60% with a 12-h light/dark cycle. All animals had free access to food and water. All procedures were approved by the Animal Care Committee of Soochow University and the behavioral tests were in accordance with the requirements of the International Association for the Study of Pain.

### Induction of Chronic Visceral Pain

Chronic visceral pain was induced by neonatal maternal deprivation (NMD) as previously described [12, 34, 35]. In brief, newborn male pups were separated from their mothers for 3 h per day from the second day after birth for 14 continuous days, while control (CON) mice did not receive any treatment. Experiments were performed when the mice were 6 weeks old.

### Electrode Implantation and Electromyographic (EMG) Recordings

To record abdominal EMG activity, electrodes (Biopac Systems, Inc, Goleta, CA) were implanted as described previously [36–38]. In brief, the mice were deeply anesthetized with isoflurane. Two sterilized nickel-chromium electrodes were implanted into the external abdominal oblique muscle using an aseptic technique and the incision was closed with a 4–0 silk suture. Visceral pain was assessed by EMG recording of colorectal distension 7 days after surgery, and traces were analyzed *via* Acknowledge (Biopac Systems, Inc).

### Visceral Pain Tests

Visceral pain was assessed by the colorectal distention (CRD) threshold and EMG as described previously [39, 40]. In brief, an inflatable balloon was made from the small finger of a small latex glove and a thin tube and then inserted from the mouse’s anus into the colorectum. The device was secured to the tail using medical-grade pressure-sensitive adhesive tape to ensure stability. Prior to testing, mice were individually placed in transparent observation chambers, and an adaptation period of 30 min–60 min was implemented to minimize stress responses. The visceral pain threshold was detected by applying different degrees of dilation pressure from 20 mmHg to 80 mmHg through a sphygmomanometer. The protocol consisted of 20-s distension steps, interspersed with 2–3 min rest periods. EMG was monitored during distension phases at different pressure levels. All behavioral experiments were carried out in a blinded manner.

### Immunofluorescence Staining

Mice were intracardially perfused with 0.9% saline and 4% paraformaldehyde in phosphate-buffered saline (PBS). Brains were postfixed at 4 °C overnight, then dehydrated in 10%, 20% and 30% sucrose. Thirty micrometer (30 µm) coronal sections were cut on a Leica CM1950 vibratome. The brain sections were restored to room temperature prior to antigen retrieval in boiling sodium citrate solution for 10 min. After three rinses in PBS for 10 min each, the sections were incubated in a blocking solution containing 7% donkey serum and 0.3% Triton X-100 in PBS for 1 h prior to overnight incubation with the primary antibodies at 4 °C. Following another 3×10 min PBS washes, the sections were incubated in appropriate secondary antibodies for 1 h at room temperature, rinsed again in PBS, and mounted in a medium containing DAPI. The primary antibodies used were rabbit anti-glutamate (1:200, rabbit, Sigma-Aldrich, USA), rabbit anti-GABA (1:500, rabbit, Sigma-Aldrich, USA), mouse anti-c-Fos (1:100, mouse, Santa Cruz Biotechnology, USA), and mouse anti-NR2A (1:20, mouse, Santa Cruz Biotechnology, USA). The secondary antibodies were Alexa Fluor 555 donkey anti-mouse (1:1000, Invitrogen, USA) and Alexa Fluor 488 donkey anti-rabbit (1:1000, Invitrogen, USA). Fluorescence signals were captured using a Zeiss Axioscope A1 microscope. For the statistical analysis of c-Fos data, we utilized ImageJ software to quantify the positive signals in the VTA. Each group comprised sections located at the same coordinates. In this portion of the experiment, a brain section was extracted from one mouse.

### Stereotaxic Injection

For virus injection, adult male mice aged 6–8 weeks were anesthetized with isoflurane and fixed on a stereotaxic apparatus (RWD, 71,000-M, Shenzhen, China). Physiological temperature (37 °C) was maintained using a feedback-controlled heating system throughout the experimental procedure. Following disinfection with iodophor, the skull was exposed *via* midline incision, and a small craniotomy was made at the predetermined stereotaxic coordinates (RWD Life Technology, China). The viruses were infused at 20 nL/min, and a volume of 200 nL viruses was injected with an infusion pump (Longer Pump, TJ-2A, Baoding, China). The micropipette was left in position for an additional 10 min to optimize viral spread and prevent reflux. To ensure adequate viral expression, behavioral testing was initiated 3 weeks after the surgical procedures.

For fiber photometry, 200 nL AAV2/9-vglut2-GCaMP6f-EGFP (BrainVTA, Wuhan, 2.94 × 10^12^ genome copies/mL) were unilaterally injected into the VTA.

To achieve precise control over glutamatergic neuronal populations in the VTA, AAV2/9-vglut2-hChR2-EGFP (BrainVTA, Wuhan, 3.0 × 10^12^ genome copies/mL), AAV2/9-vglut2-eNpHR-EGFP (BrainVTA, Wuhan, 2.35 × 10^12^ genome copies/mL), and AAV2/9-vglut2-hM3Dq-mCherry (BrainVTA, Wuhan, AAV2/9, 5.00 × 10^12^ genome copies/mL) were administered by unilateral injection.

### Fiber Photometry System

Mice were unilaterally injected with AAV2/9-vglut2-GCaMP6f (BrainVTA, Wuhan, 2.94 × 10^12^ genome copies/mL) into the right VTA (in mm: anterior posterior (AP), −3.28; medial lateral (ML), −0.1; dorsal ventral (DV), −4.3). After the viruses were injected, a fiber optic cannula was implanted into the VTA (AP, −3.28; ML, −0.1; DV, −4.2). The fiber photometry system (ThinkerTech Nanjing Bioscience Inc.) consisted of 470 nm and 405 nm channels with the unique 405 nm as a reference channel to effectively remove interference [41–43]. We plotted changes in the fluorescence of GCaMP6f representing the Ca^2+^ activity of glutamatergic VTA neurons in response to CRD stimulation at 20, 40, 60, and 80 mmHg. We derived the value of the fluorescence change by calculating *ΔF/F0*, where *ΔF* is the difference in fluorescence between each sample and F0 is the average fluorescence baseline over the entire test period (0s–10s). Nine trials of repeated CRD stimulation were applied during Ca^2+^ signal recording. Photometry data were exported to MatLab R2021a for further analysis.

### Optogenetic Manipulation

For optogenetic inhibition of glutamatergic VTA neurons, AAV2/9-vglut2-eNpHR-EGFP (BrainVTA, Wuhan, 2.35 × 10^12^ genome copies/mL) was unilaterally injected into the VTA. For optogenetic activation of glutamatergic VTA neurons, AAV2/9-vglut2-hChR2-EGFP (BrainVTA, Wuhan, 3.0 × 10^12^ genome copies/mL) and AAV2/9-vglut2-EGFP (BrainVTA, Wuhan, 2.25 × 10^12^ genome copies/mL) were unilaterally injected into the VTA of CON mice. After the viruses were injected, an optical fiber was implanted into the VTA. For optogenetic inhibition or activation of the VTA, EMG was recorded during the delivery of a 20-s pulse of yellow (594 nm, 3 mW–5 mW, constant) or blue light (473 nm, 2 mW–5 mW, 20-ms pulses, 10 Hz) controlled by STSI-Optogenetics-LED (Alpha Omega Engineering, Nazareth, Israel). Each mouse was perfused and sections were cut to detect the fluorescence expression of the viruses after all the experiments.

### Chemogenetic Manipulation

To investigate the regulatory relationship between NR2A in glutamatergic neurons with glutamate neurons, AAV2/9-vglut2-hM3Dq-mCherry (BrainVTA, Wuhan, 5.00×10^12^ genome copies/mL) was unilaterally injected into the VTA of NMD and CON mice.

### Drug Administration

To verify the modulatory effect of NR2A on visceral pain, normal saline (NS) and the NR2A antagonist NVP-AAM077 (MedChemExpress, USA; 1, 10, and 100 µmol/L; 1µL) were microinjected into the VTA of NMD mice *via* the implanted cannula. In addition, Clozapine N-oxide (CNO, BrainVTA, Wuhan; 0.33 mg/mL) was administered *via* the implanted cannula.

### Real-time Quantitative Polymerase Chain Reaction (RT-qPCR)

RT-qPCR was used to assess the mRNA level of NR2A in the VTA of CON and NMD mice. In brief, triazole (TransGen Biotech) was used to extract total mRNA from VTA tissue, and mRNA concentration was determined by a Nanodrop spectrophotometer. According to the ratio in the reverse transcription kit (TransGen Biotech), the reagent was mixed, and the reverse transcription reaction was carried out by a PCR (Bio-Rad) instrument with the reaction conditions 85 °C for 5 s, 42 °C for 15 min, and 4 °C to end. The mRNA in the VTA was reverse-transcribed into cDNA. Then, the prepared primers (Table 1), components of the PerfectStart Green qPCR SuperMix (+DyeII), and reverse transcription cDNA were added into a centrifuge tube and placed in the qPCR instrument (ABI-7500, USA) with the reaction conditions 1 cycle at 95 °C for 10 min, 40 cycles at 95 °C for 15 s, and 60 °C for 45 s for reaction. After the reaction, the Ct value of the sample was read and analyzed using the comparative Ct method [44, 45], based on the formula: relative mRNA expression = 2^−(Ct of target gene – Ct of GAPDH)^ [44]. In order to ensure the reliability of the data, each sample was repeated 3 times.

**Table 1:** Primer sequences used in the present study.

Primers Sequence (5’ to 3’)	
GAPDH-F	GAAGGTCGGTGTGAACGGAT
GAPDH-R	AATCTCCACTTTGCCACTGC
NR2A-1F	AAGAAGTGCTGCAAGGGGTT
NR2A-1R	GCCCTTCTGCAGAGCAATTC
NR2B-1F	TCGGCCAAATCTAGGAGGGA
NR2B-1R	CCCCAGACCTATCCTCCCAA
NR2C-1F	GGAGGAATACGACTGGAGCG
NR2C-1R	GGCCAGCTTCTTGAGGATGT
NR1-1F	CACAATTACGAGAGCGCAGC
NR1-1R	GGCCCTCCTCCCTCTCAATA
GluR1-1F	GGTTGTGGGTGCCAATTTCC
GluR1-1R	ACTGCTGCATGATTCTGGCT
GluR2-1F	CCACCTACCCTGATGTGTCTTT
GluR2-1R	TGGGTAAGGTGGTAGAGCGT
GluR3-1F	AACACGGTACAGGAGCACAG
GluR3-1R	CCCAGCGCTGTATGAATTGC
GluR4-1F	AGGGATGCGGACACCAAAAT
GluR4-1R	CGCTGCCACATTCTCCTTTG

### Western Blotting

VTA tissue was added to a centrifugal tube containing cell lysate and protease inhibitors (Sigma) and homogenized by an ultrasonic crusher. The protein concentration was determined with a BCA kit. A total of 20 µg of supernatant was placed in a new centrifuge tube, and dissolved in a uniform volume of saline. The protein solution was added to the polyacrylamide gels of 10% sodium dodecyl sulphate for gel electrophoresis (Bio-Rad) and then transferred to polyvinylidene difluoride membranes (Merck Millipore) at 200 mA for 2 h. The membranes were placed in a 5% fat-free milk-blocking solution at room temperature for 2 h prior to 24 h incubation with the primary antibody (anti-GAPDH, 1:1000, GoHere Technology, AB-P-R 001 and anti-NR2A, 1:2000, Sigma, ZRB04091) at 4 °C. Following washes with TBST (15 min × 3), the membranes were incubated with the secondary antibody at room temperature for 2 h, washed again (15 min × 3), and visualized using an enhanced chemiluminescence detection kit for HRP (EZ-ECL, Biological Industries 20-500-120) and the chemiluminescence imaging system (ChemiDoc XRS, Bio-Rad, Hercules, CA, USA). The densities of bands were measured using ImageJ software. Protein expression was normalized to GAPDH.

### Statistical Analyses

All data are reported as the mean ± SEM; error bars indicate the SEM. Statistical analysis was performed using GraphPad Prism software. Data points deviating from the mean by >0.2 standard deviations were defined as outlier data. The data were assessed by one-way ANOVA, two-way ANOVA followed by Sidak’s multiple comparisons test, or two-sample Student’s *t*-test. *P* <0.05 was considered a statistically significant difference.

## Results

### NMD Induces Visceral Pain and Activates Glutamatergic VTA Neurons in Mice

**Fig. 1:**
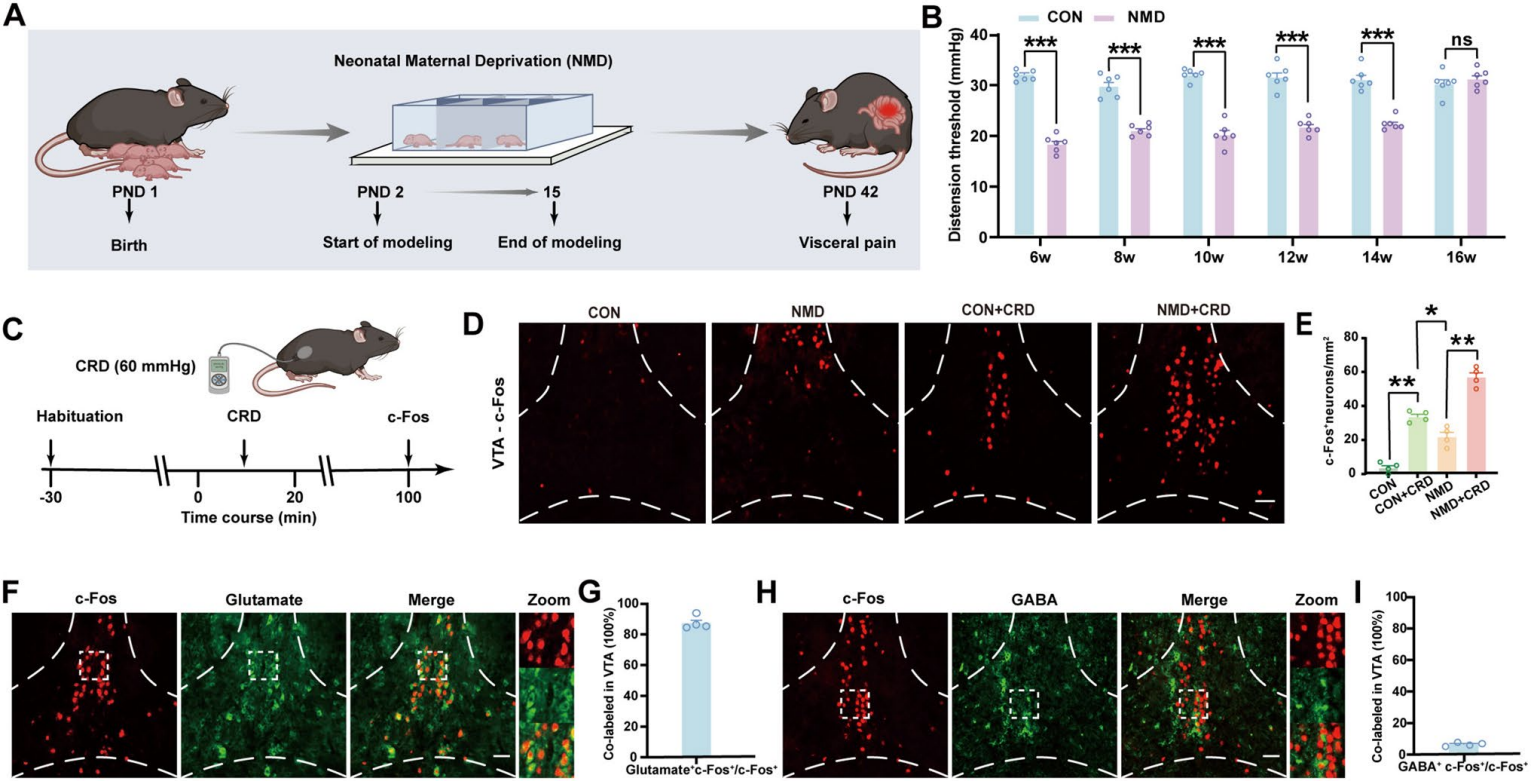
The expression of c-Fos in the VTA is significantly increased upon visceral pain stimulation. **A** Experimental protocol for NMD model preparation. **B** Visceral pain thresholds in CON and NMD mice from 6 to 16 weeks (***P <0.001, two-way ANOVA followed by Sidak’s multiple comparisons test, *n =* 6 mice per group). **C** Schematic of the experimental procedure for inducing c-Fos expression through CRD stimulation. **D** Representative images and quantification of c-Fos-positive cell immunofluorescence in the VTA of CON and NMD mice (scale bar, 100 µm, one-way ANOVA with Tukey’s multiple comparisons test, *n =* 4 brain sections from 4 mice per group). **F** Representative images of glutamatergic neurons and c-Fos-positive cells in the VTA (scale, 100 µm). **G** The ratio of glutamate and c-Fos co-labeled neurons in total c-Fos-positive neurons (*n =* 4 sections from 4 mice per group). **H** Representative immunofluorescence images of GABAergic neurons and c-Fos-positive cells in the VTA (scale bar, 100 µm). **I** Percent of GABA and c-Fos co-labeled neurons in total c-Fos positive neurons (*n =* 4 sections from 4 mice per group). ns, no significant difference, *P* >0.05.

A schematic of the NMD mouse model is shown in Fig. 1A. The NMD mouse produced significant visceral pain compared with CON mice (Fig. 1B, ***P <0.001, two-way ANOVA followed by Sidak’s multiple comparisons test), which is consistent with our previous study [12, 46–48]. This result confirmed that visceral pain was successfully induced in adult mice. To test whether the VTA is activated by visceral pain, mice were given CRD stimulation (Fig. 1C), and c-Fos expression increased dramatically after repeated CRD stimulation, particularly in the VTA. In addition, c-Fos expression was more markedly increased in the VTA of NMD mice than in CON mice (Fig. 1D and E, *P <0.05, **P <0.01, one-way ANOVA with Tukey’s multiple comparisons test).

To further investigate the types of c-Fos-positive cells activated in the VTA, immunofluorescence was assessed. Immunostaining revealed that c-Fos-positive cells were extensively co-expressed with the glutamatergic neurons (Fig. 1F). The ratio of glutamate^+^ + c-Fos^+^ / c-Fos^+^ was 87.17% (Fig. 1G). In addition, we also assessed the co-expression of c-Fos-positive cells with GABAergic neurons (Fig. 1H). The ratio of GABA^+^ + c-Fos^+^ / c-Fos^+^ was 5.76 % (Fig. 1I). It was therefore evident that the majority of c-Fos-positive cells are glutamatergic neurons in the VTA of NMD mice, suggesting that these neurons play an important role in NMD-induced chronic visceral hypersensitivity.

### Visceral Pain Stimulation Significantly Enhances the Ca^2+^ Activity of Glutamatergic VTA Neurons

**Fig. 2:**
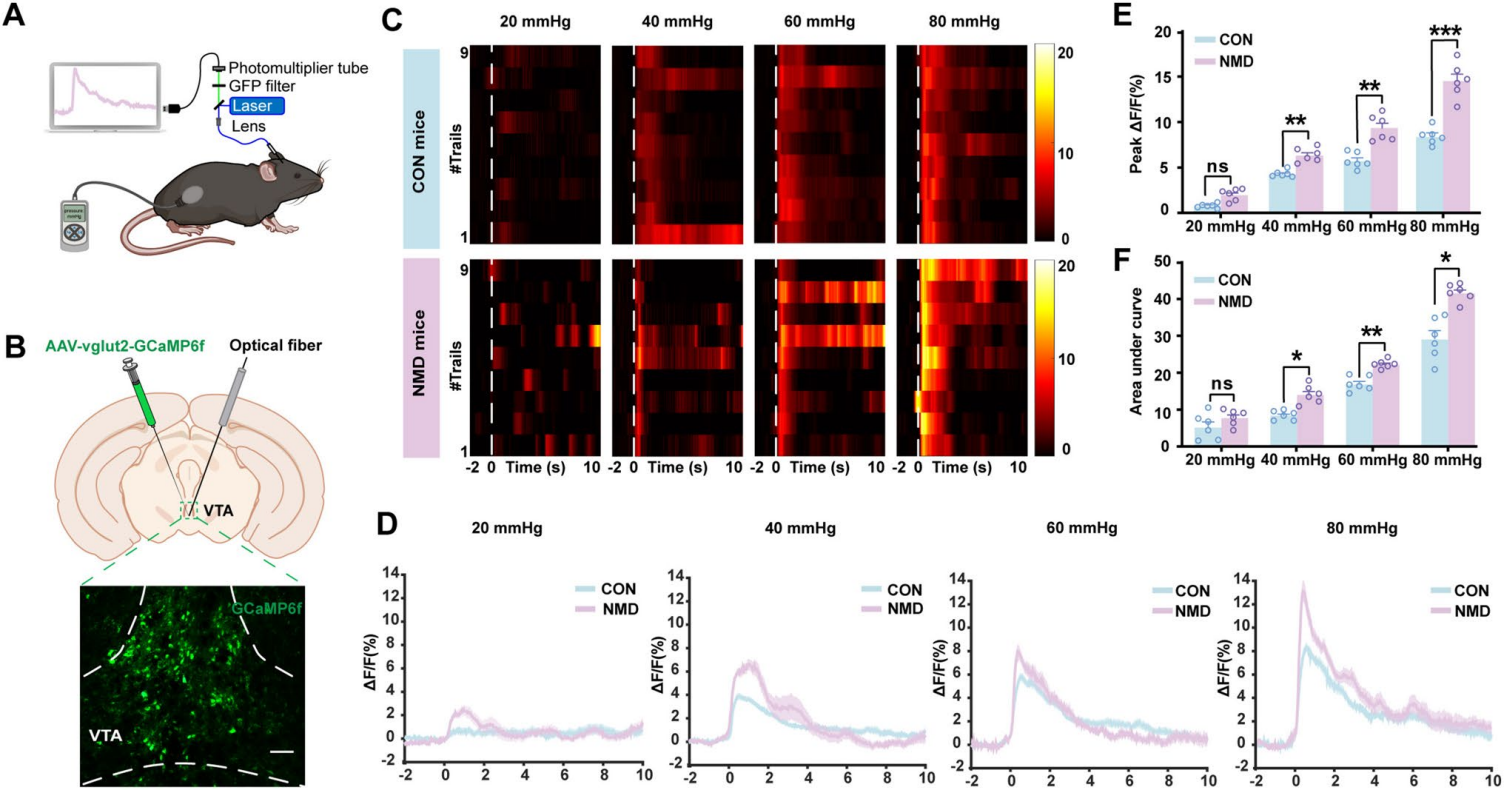
Visceral pain stimulation induces a dramatic increase in Ca^2+^ activity of glutamatergic VTA neurons. **A** Cartoon of a recording system for assessing Ca^2+^ signals utilizing fiber photometry and visceral pain behavior induced by CRD stimulation. **B** Upper, placement of injector and optical fiber; lower, representative fluorescence image of AAV-vglut2-GCaMP6f viral expression in the VTA (scale bar, 100 µm). **C**, **D** Heatmaps and average Ca^2+^ transients of fiber photometry recordings in response to 20, 40, 60, and 80 mmHg CRD stimulation in CON and NMD mice. **E** Average peak values of Ca^2+^ activity of glutamatergic VTA neurons in CON and NMD mice receiving 20, 40, 60, and 80 mmHg stimulation (**P <0.01, ***P <0.001, two-way ANOVA followed by Sidak’s multiple comparisons test, *n =* 6 per group). **F** Area under the curve of glutamatergic VTA neurons in CON and NMD mice receiving 20, 40, 60, and 80 mmHg stimulation (*P <0.05,**P <0.01, two-way ANOVA followed by Sidak’s multiple comparisons test, *n =* 6 per group). ns, no significant difference, *P* >0.05.

To further determine the types of neurons activated in the VTA during visceral stimulation, fiber photometry was applied to record the Ca^2+^ signal changes *in vivo* from these neurons in awake behaving mice (Fig. 2A). AAV2/9-vglut2-GCaMP6f was stereotactically injected into the VTA to infect glutamatergic neurons (Fig. 2B). As shown in Fig. 2C and D, the Ca^2+^ signals of glutamatergic VTA neurons exhibited sharp fluctuations during 40 mmHg, 60 mmHg, and 80 mmHg stimulation. However, the Ca^2+^ activity of NMD mice was significantly higher than that of CON mice (Fig. 2E and F, *P <0.05, **P <0.01, ***P <0.001, two-way ANOVA followed by Sidak’s multiple comparisons test). These results suggested that VTA glutamatergic neurons are more dramatically activated by visceral pain stimulation, which is consistent with our previous data from the c-Fos test.

### Optogenetic Manipulation of Glutamatergic VTA Neurons Alters Visceral Pain

**Fig. 3:**
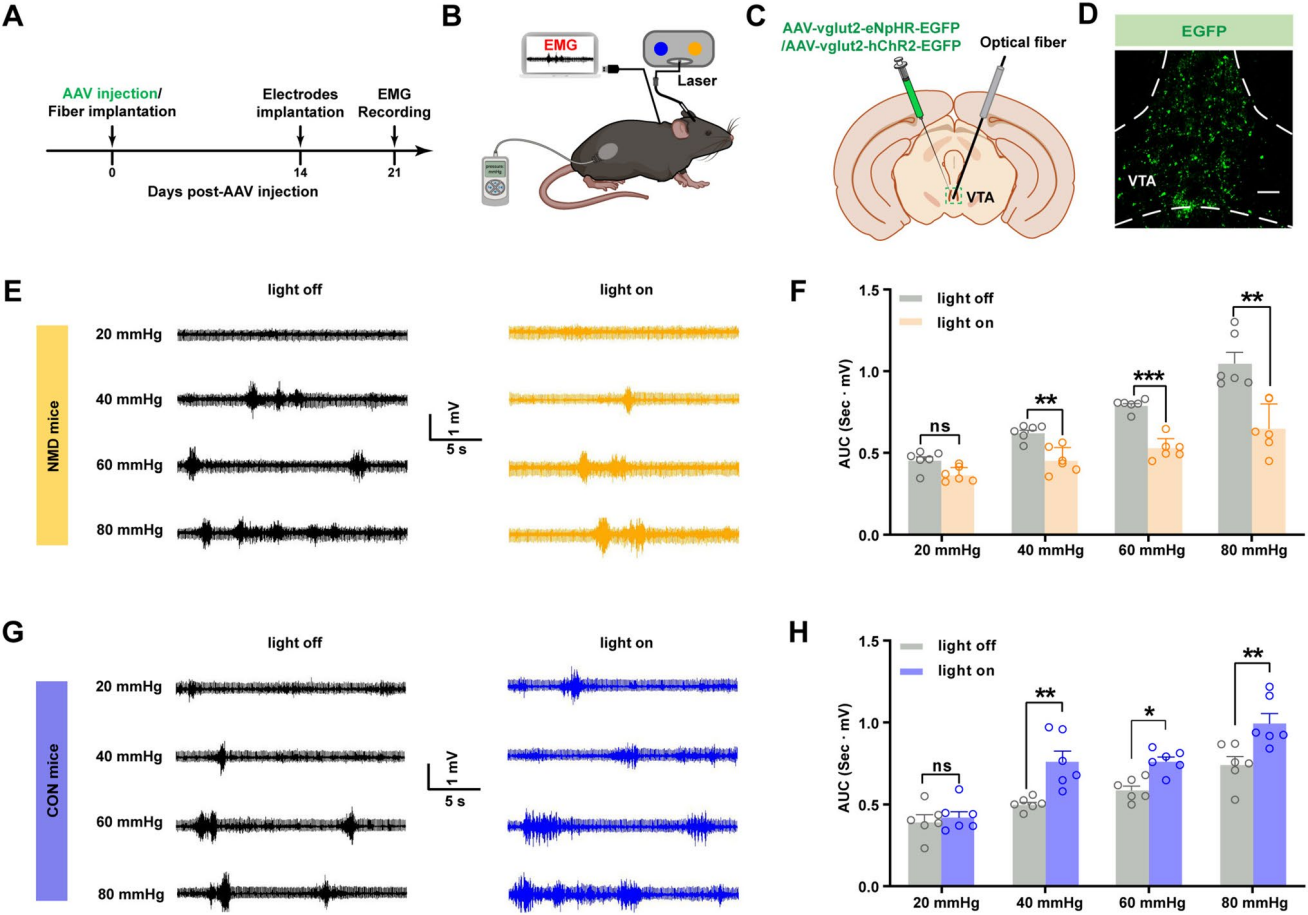
Optogenetic inhibition of glutamatergic VTA neurons alleviates visceral pain in NMD mice, whereas their activation leads to visceral pain in CON mice. **A** Flowchart of optogenetic manipulation of glutamatergic VTA neurons in NMD and CON mice. **B** Cartoon of EMG recording for assessing visceral pain. **C** Schematic of virus injection and optical fiber implantation in the VTA. **D** Representative image of virus expression in glutamatergic VTA neurons (scale bar, 100 μm). **E** Representative EMG traces of NMD mice at different levels of CRD stimulation (20, 40, 60, and 80 mmHg). **F** The area under the curve of the EMG in NMD mice at 20, 40, 60, and 80 mmHg (**P <0.01, ***P <0.001, two-way ANOVA followed by Sidak’s multiple comparisons test, *n =* 6 per group). **G** Representative EMG traces of CON mice at different levels of CRD stimulation (20, 40, 60, and 80 mmHg). **H** The area under the curve of the EMG in CON mice at 20, 40, 60, and 80 mmHg (*P <0.05, **P <0.01, two-way ANOVA followed by Sidak’s multiple comparisons test, *n =* 6 per group). ns, no significant difference, *P* >0.05. See also Figs. S1 and S2.

In order to further investigate the regulatory role of glutamatergic VTA neurons in chronic visceral pain, optogenetics was applied to selectively manipulate these neurons and evaluate visceral pain by CRD combined with EMG recording (Fig. 3A, B). AAV2/9-vglut2-hChR2-EGFP, AAV2/9-vglut2-eNpHR-EGFP, and AAV2/9-vglut2 -EGFP were injected into the VTA to infect glutamatergic neurons (Figs. 3C, D; S1C, D; and S2C, D). Opto-silencing of glutamatergic VTA neurons significantly decreased the visceral pain responses in NMD mice (Fig. 3E, F; **P <0.01, ***P <0.001, two-way ANOVA followed by Sidak’s multiple comparisons test), whereas activation of these neurons increased visceral pain responses in CON mice (Fig. 3G, H; *P <0.05, **P <0.01, two-way ANOVA followed by Sidak’s multiple comparisons test). However, neither inhibition nor activation of control viral-infected glutamatergic VTA neurons altered visceral pain in NMD (Fig. S1) or CON mice (Fig. S2). These results suggested that VTA glutamatergic neurons modulate visceral pain.

### Expression of NR2A is Significantly Upregulated in the Glutamatergic VTA Neurons of NMD Mice

**Fig. 4:**
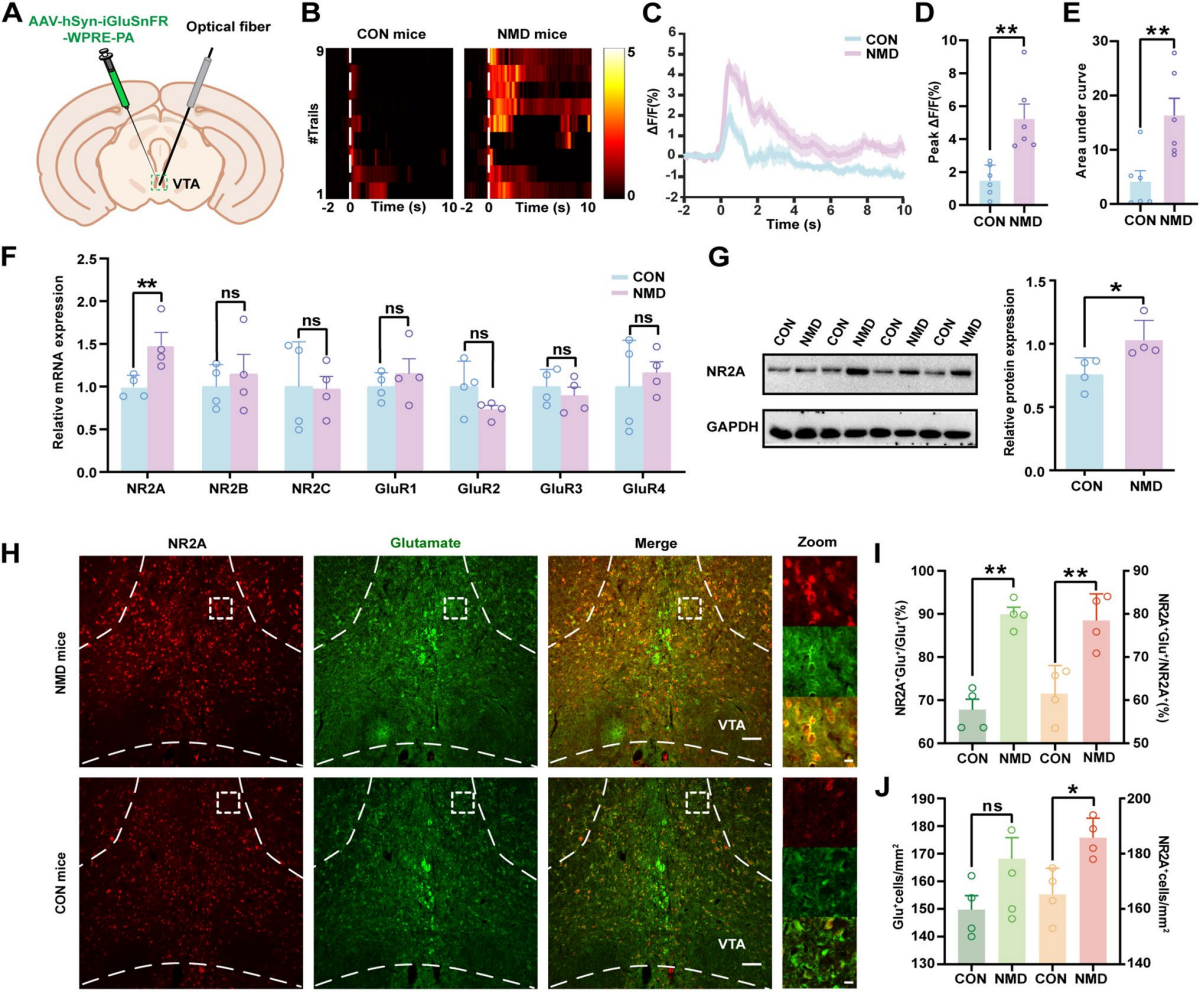
Increased Glutamate Release and Upregulation of NR2A Expression in the VTA of NMD Mice. **A** Schematic of glutamate sensor injection and optical fiber implantation in the VTA. **B**, **C** Heatmaps and waveform plots of fiber photometry recordings in response to CRD stimulation at 60 mmHg. **D** Average peak for glutamate signals of glutamatergic VTA neurons while CON and NMD mice receive CRD stimulation (**P <0.01, two-sample *t*-test, *n =* 6 per group). **E** The area under the curve for glutamate signals of glutamatergic VTA neurons while CON and NMD mice receive CRD stimulation (**P <0.01, two-sample *t*-test, *n =* 6 per group). **F** mRNA expression levels of NMDARs and the GluR1-4 subunits of AMPARs in the VTA (**P <0.01, two-sample *t*-test, *n =* 4 per group). **G** The protein patterns of NR2A in the VTA and quantification of changes of NR2A protein expression in the VTA (*P <0.05, two-sample *t*-test, *n =* 4 per group). **H** Representative images for the co-localization of NR2A (red) and glutamatergic neurons (green) in the VTA of NMD and CON mice (Scale bar, 100 μm). **I** Percentage of glutamatergic neurons among NR2A^+^ neurons in CON and NMD mice, and the ratio of NR2A^+^ + Glu^+^ / NR2A^+^ in the VTA (**P <0.01, two-sample *t*-test, *n =* 4 sections from 4 mice per group). **J** The density of glutamatergic neurons and the number of NR2A^+^neurons in the VTA of CON and NMD groups (*P <0.05, two-sample *t*-test, *n =* 4 sections from 4 mice per group). ns, no significant difference, *P* >0.05.

The above data demonstrated that activation of glutamatergic VTA neurons is a key factor in the development of visceral pain behavior in NMD mice. To investigate the mechanism by which these neurons are activated, we tested changes in glutamate release in the VTA during visceral pain stimulation *via* a glutamate sensor (Fig. 4A). This showed that glutamate release in the VTA of NMD mice was dramatically increased upon CRD stimulation compared to CON mice (Fig. 4B, C, D, E; **P <0.01, two-sample Student’s t-test). Therefore, we further examined the expression of NMDARs and the GluR1-4 subunits of AMPARs in the VTA. Compared to the CON mice, NR2A mRNA expression was significantly upregulated in the VTA of NMD mice, whereas there were no significant differences in the expression of NR2B, NR2C, GluR1, GluR2, GluR3, and GluR4 (Fig. 4F; **P <0.01, two-way repeated-measures ANOVA followed by Sidak’s multiple comparisons test). Furthermore, the upregulation of NR2A protein expression in the VTA of NMD mice was confirmed by western blot (Fig. 4G; *P <0.05, two-sample Student’s *t*-test).

In addition, immunofluorescence results showed that the expression of NR2A in the glutamatergic VTA neurons of NMD mice was significantly upregulated compared to CON mice and that glutamatergic neurons were the primary co-localization unit of NR2A (Fig. 4H, I, J; *P <0.05, **P <0.01, two-sample *t*-test). These findings suggested that the selective upregulation of NR2A in VTA might underlie the development of visceral pain in NMD mice.

### Injection of the NR2A Antagonist NVP-AAM077 into the VTA Alleviates Visceral Pain in NMD Mice

**Fig. 5:**
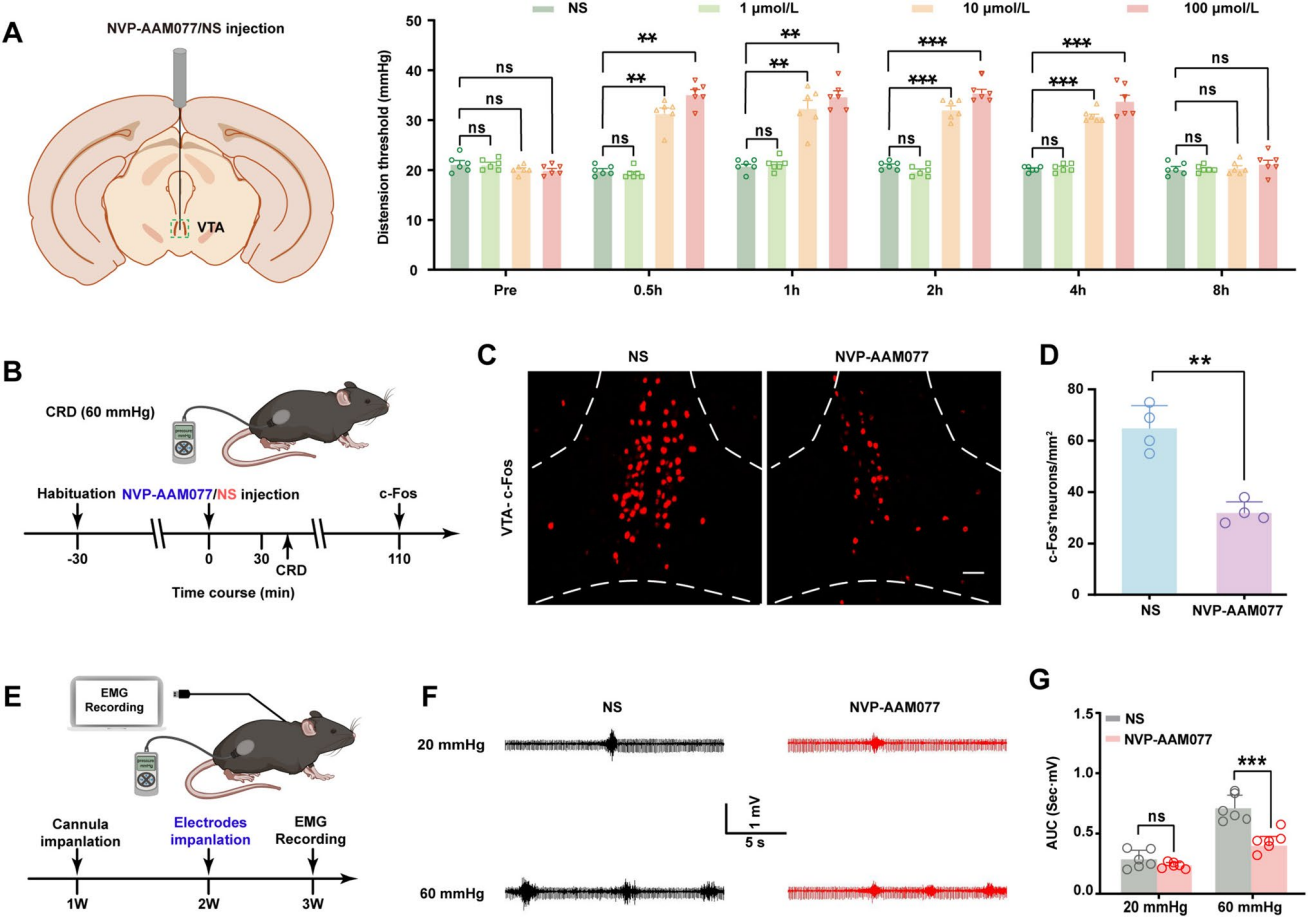
Injection of NVP-AAM077 inhibits VTA activation and alleviates visceral pain. **A** Effects of different doses of NVP-AAM077 on visceral pain thresholds in NMD mice (**P <0.01, ***P <0.001, two-way repeated-measures ANOVA followed by Sidak’s multiple comparisons test, *n =* 6 per group). **B** Experimental time course for injection of NS or NVP-AAM077 into the VTA of NMD mice followed by CRD stimulation and tissue harvest for c-Fos immunofluorescence. **C** Representative c-Fos images in the VTA of NMD mice following injection of NS or NVP-AAM077 (scale bar, 100 μm). **D** The numbers of c-Fos-positive cells in the VTA of NS and NVP-AAM077 groups (**P <0.01, two-sample *t*-test, *n =* 3 per group). **E** Flowchart of EMG signal restoration procedure. **F** Representative EMG traces in response to 20 and 60 mmHg CRD stimulation in NMD mice injected with NVP-AAM077 (red) or NS (black) in the VTA. **G** The AUC of EMG during CRD stimulation following injection of NVP-AAM077 or NS into the VTA of NMD mice (***P <0.001, two-way repeated-measures ANOVA followed by Sidak’s multiple comparisons test, *n =* 6 per group). ns, no significant difference, *P* >0.05.

We applied pharmacological experiments to investigate the effect of upregulation of NR2A expression in the VTA of NMD mice with visceral pain. To determine the most appropriate concentration, a cannula was implanted into the VTA of NMD mice utilizing stereotaxic techniques, and then 1 µL of NS or the NR2A antagonist NVP-AAM077 (1 µmol/L, 10 µmol/L, or 100 µmol/L) was injected into the VTA using a microsyringe pump (Fig. 5A). Compared to the NS group, 1 µmol/L of NVP-AAM077 did not reverse the visceral pain threshold in NMD mice, but injections of 10 µmol/L or 100 µmol/L of NVP-AAM077 significantly increased their threshold (Fig. 5A; **P <0.01, ***P <0.001, two-way repeated-measures ANOVA followed by Sidak’s multiple comparisons test). Therefore, 10 µmol/L of NVP-AAM077 was selected for subsequent experiments. To assess the effect on VTA neuronal activity following NVP-AAM077 injection (Fig. 5B), immunostaining was applied to test c-Fos expression (Fig. 5C). NVP-AAM077 decreased the density of c-Fos-positive cells in the VTA of NMD mice (Fig. 5C, D; **P <0.01, two-sample Student’s t-test), suggesting that NR2A is a critical factor in activating VTA neurons. In addition, EMG recordings confirmed that NVP-AAM077 (10 µmol/L) provided significant relief of visceral pain responses in NMD mice (Fig. 5E, F, G; ***P <0.001, two-way repeated-measures ANOVA followed by Sidak’s multiple comparisons test). These results indicated that NR2A in the VTA regulates chronic visceral pain in NMD mice.

### Upregulation of NR2A Contributes to Visceral Pain in NMD Mice by Hyperactivating Glutamatergic VTA Neurons

**Fig. 6:**
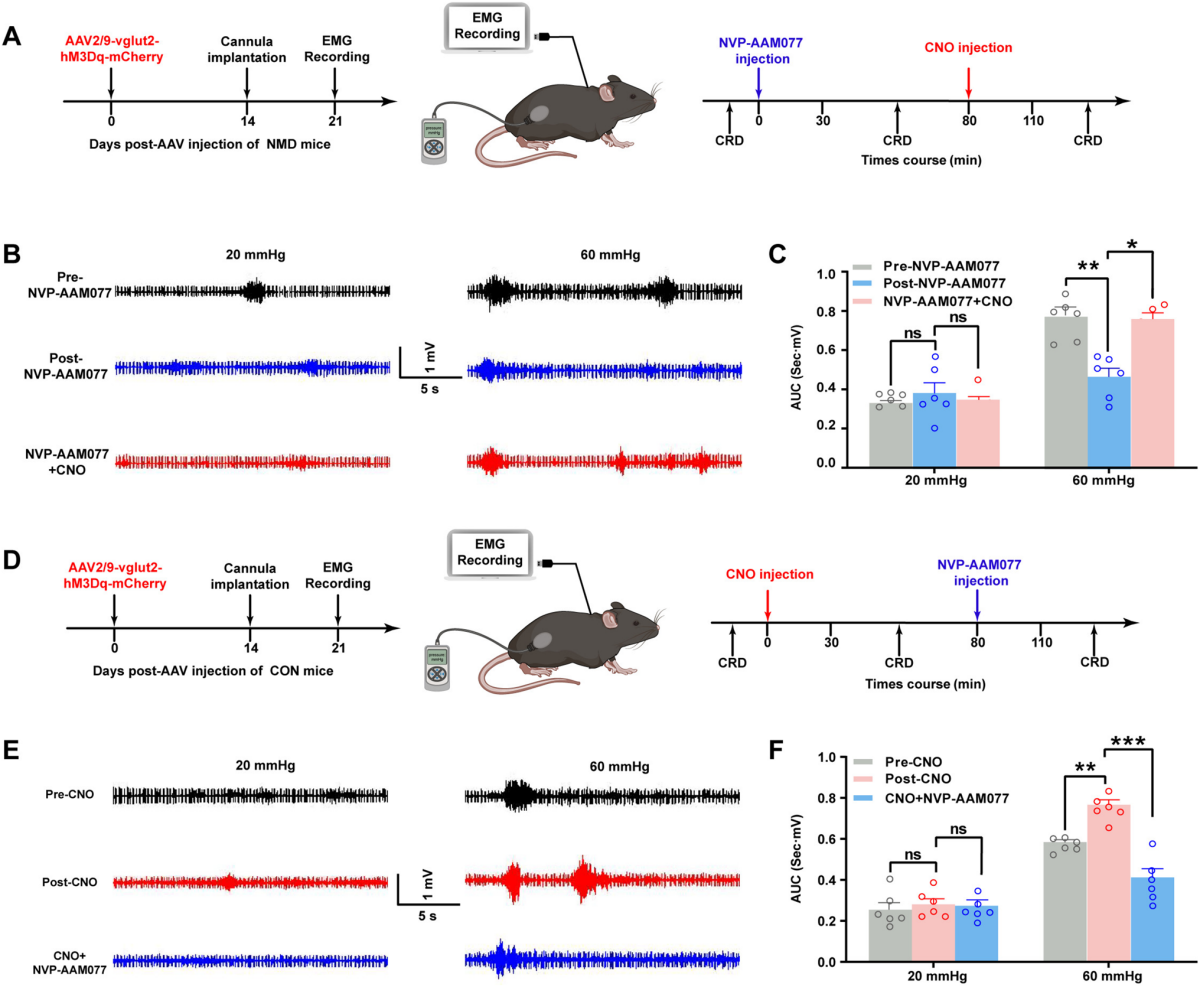
NR2A contributes to visceral pain *via* activation of glutamatergic VTA neurons. **A** Experimental procedure of chemogenetics combined with pharmacology in NMD mice. **B** Representative EMG traces of pre-NVP-AAM077, post-NVP-AAM077, and NVP-AAM077 + CNO groups. **C** The area under the curve of EMG at 60 mmHg in NMD mice responding to pre-NVP-AAM077, post-NVP-AAM077, and NVP-AAM077 + CNO groups (*P <0.05, **P <0.01, two-way ANOVA followed by Sidak’s multiple comparisons test, *n =* 6 per group). **D** Schematic of the procedure of chemogenetics combined with pharmacology in CON mice. **E** Representative EMG traces of pre-CNO, post-CNO, and CNO + NVP-AAM077 groups. **F** The area under the curve of EMG at 60 mmHg in CON mice responding to pre-CNO, post-CNO, and CNO + NVP-AAM077 groups (**P <0.01, ***P <0.001, two-way ANOVA followed by Sidak’s multiple comparisons test, *n =* 6 per group). ns, no significant difference. *P* >0.05.

**Fig. 7:**
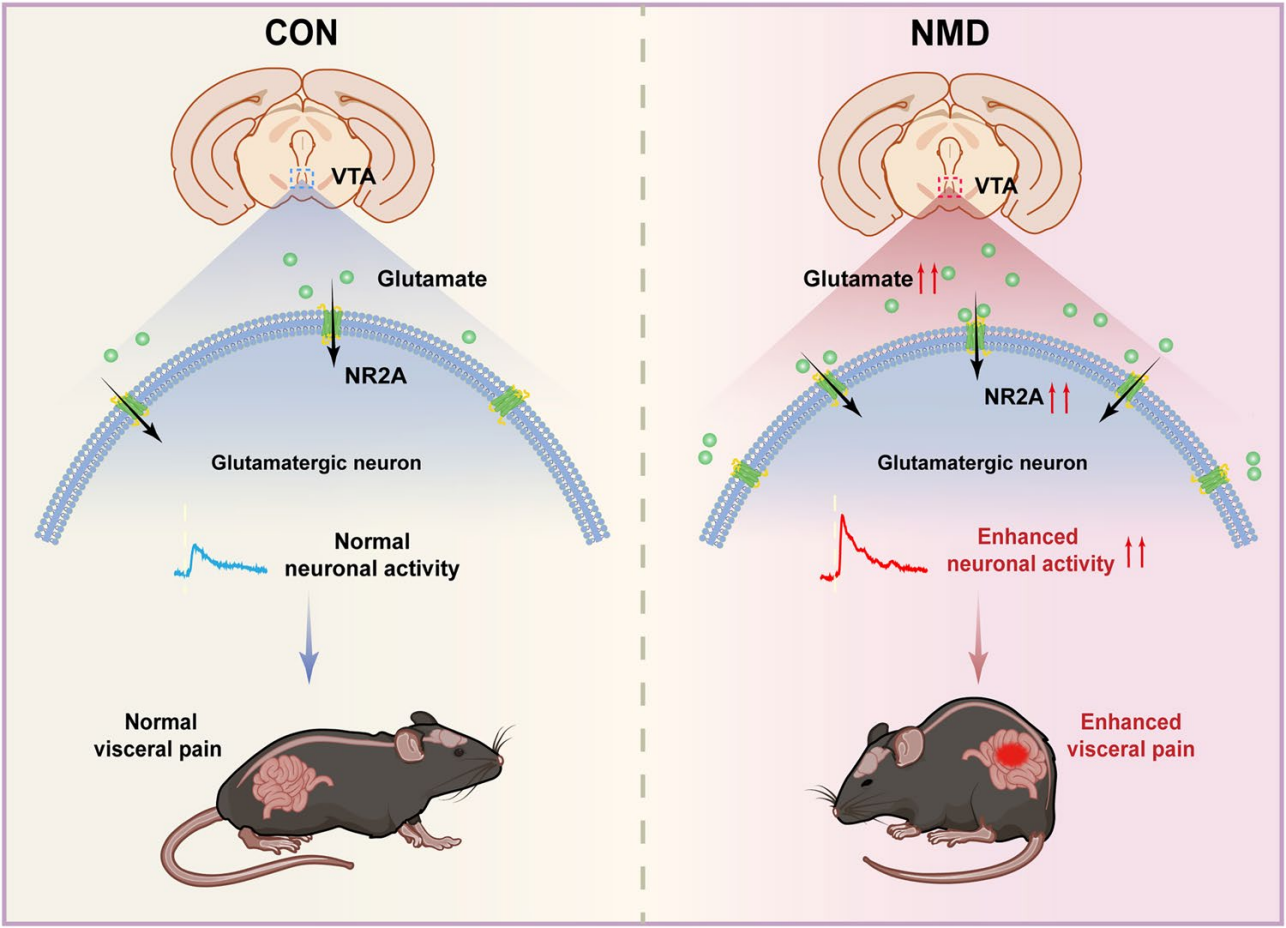
Schematic Model: NR2A in Glutamatergic VTA Neurons Regulates Chronic Visceral Pain in Male Mice.

We used chemogenetic and pharmacological techniques to further validate the contribution of NR2A-mediated hyperactivation of glutamatergic VTA neurons to visceral pain. The results showed that the application of NVP-AAM077 (10 µmol/L) significantly alleviated the visceral pain response in NMD mice, whereas this alleviation was blocked by the chemogenetic activation of glutamatergic VTA neurons (Fig. 6A, B, C; *P <0.05, **P <0.01, two-way ANOVA followed by Sidak’s multiple comparisons test). In addition, activation of glutamatergic VTA neurons markedly increased visceral pain in CON mice, while this reinforcing effect was blocked by NVP-AAM077 (Fig. 6D, E, F; **P <0.01, ***P <0.001, two-way ANOVA followed by Sidak’s multiple comparisons test). These results demonstrated that upregulated NR2A induces visceral pain in NMD mice through hyperactivation of glutamatergic VTA neurons, suggesting that upregulation of NR2A is necessary and sufficient for the development of visceral pain in NMD mice (Fig. 7).

## Discussion

Chronic visceral pain is a predominant factor driving individuals with IBS to seek medical intervention. However, the incomplete understanding of the central mechanisms underlying chronic visceral pain has significantly hindered the development of effective therapeutic strategies. In this study, we demonstrate that glutamatergic neurons in the VTA play a crucial role in mediating visceral pain in NMD mouse models. Furthermore, we identify the NMDAR subtype NR2A as a critical target influencing behavioral responses to visceral pain. These findings provide a foundation for further understanding of the neural circuits and molecular targets underlying such pain, offering a theoretical basis for developing targeted therapies to alleviate this condition.

The VTA comprises dopaminergic, GABAergic, and glutamatergic neurons. This region is known to play a part in a wide range of behaviors, including pain, aversion, reward, learning, and memory, and is also implicated in conditions such as Parkinson’s disease and addiction [49–54]. Research has demonstrated that dopaminergic neurons in the VTA exhibit both anatomical and functional heterogeneity, serving diverse roles in pain perception [55–57]. Specifically, these neurons display differential responses to nociceptive stimuli. For instance, while some dopaminergic neurons exhibit inhibitory responses to pain stimulation, others show excitatory responses [54, 56]. Notably, vesicular glutamate transporter-2 expression has been reported in glutamatergic neurons located near the midline of the VTA, where glutamatergic neurons are more abundant than dopaminergic neurons [58, 59]. This suggests a potential modulatory function of glutamatergic VTA neurons in pain regulation. In the present study, we provide substantial evidence that visceral pain stimulation significantly increases the Ca^2+^ activity of glutamatergic VTA neurons. In addition, optogenetic manipulation of these neurons markedly alters visceral pain responses in mice, demonstrating that glutamatergic VTA neurons are key mediators in the development of visceral pain. Importantly, our findings align with similar studies [23, 60]. This investigation elucidates the critical mechanisms by which glutamatergic neurons in the VTA regulate chronic visceral pain, potentially paving the way for novel therapeutic strategies to benefit patients suffering from such conditions.

Previous studies have established that NMDARs play a crucial role in neuroplasticity, and their activation typically enhances synaptic transmission and cellular hyperexcitability [61–63]. At the spinal cord level, NMDARs amplify the response of dorsal horn sensory neurons to noxious stimuli, contributing to hyperalgesia. In addition, emerging evidence highlights the role of epitranscriptomic regulation of NMDARs in chemotherapy-induced neuropathic pain [64, 65]. In a pain model induced by hind paw injection of formalin, the upregulation of NR2A and NR2B subunits of NMDARs in the anterior cingulate cortex was found to be associated with generated pain [66]. These findings indicate the significant role of NMDARs in regulating neuronal excitability and pain development. However, evidence regarding the modulation of pain by NMDARs in the VTA remains limited. In the present study, our data strongly support the hypothesis that the NMDAR subtype NR2A regulates chronic visceral pain. We showed that NR2A was specifically upregulated in the VTA of NMD mice, and that pharmacological inhibition of NR2A reduced the excitation of glutamatergic VTA neurons, thereby alleviating visceral pain. These findings suggest that NR2A plays a significant modulatory role in visceral pain and represents a highly promising therapeutic target. Remarkably, although we demonstrated that NR2A elicits visceral pain through the activation of glutamatergic VTA neurons, a potential role for dopaminergic neurons cannot be completely ruled out, as NR2A is not exclusively expressed in glutamatergic neurons. Therefore, whether NR2A also activates dopaminergic neurons remains to be further explored, perhaps as a potential mechanism contributing to chronic visceral pain.

While we have demonstrated the role of glutamatergic VTA neurons in chronic visceral pain, the specific neural circuits through which these neurons regulate such pain remain incompletely understood. Interestingly, previous studies have shown that virtually all neurons in the medial nucleus accumbens, lateral habenula, ventral pallidum, and amygdala receive projections from glutamatergic VTA neurons [67–70]. This suggests the existence of a potential neural circuitry involved in visceral pain regulation. It is important to note that this study was conducted exclusively in male mice, and the experimental results may not be completely suitable for female mice. Previous studies have shown that estrogen and the physiological cycle of female mice significantly influence visceral pain [71–73]. Therefore, male mice were used in this study to minimize the influence of estrogen on visceral pain and to ensure more consistent results. Although our findings robustly demonstrate that glutamatergic VTA neurons play a key role in NMD-induced chronic visceral pain, the present study has certain limitations that should be addressed in future research. First, while the NMD mouse model effectively simulates the chronic visceral pain behaviors of IBS patients, it may not fully capture all aspects of the condition. Second, this study primarily focused on the role of the VTA in visceral pain behavior, but the associated neural circuits remain unclear. Future research utilizing multiple IBS models and exploring VTA-mediated neural circuits in greater depth will help provide a more comprehensive understanding of the underlying neural and molecular mechanisms.

In conclusion, this study reveals that upregulation of NR2A enhances the excitability of glutamatergic VTA neurons, thereby contributing to chronic visceral pain in mice. These findings are expected to advance our understanding of the pathogenesis of IBS from the perspective of central nervous system sensitization and provide new therapeutic targets for the development of treatments for chronic visceral pain.

## Supplementary Information

Below is the link to the electronic supplementary material.

Supplementary file1 (PDF 401 KB)
