# Supplementary material for: Upregulation of NR2A in Glutamatergic VTA Neurons Contributes to Chronic Visceral Pain in Male Mice
Source: Neurosci Bull. 2025 Apr 28;41(12):2113–26. doi: 10.1007/s12264-025-01402-7 (PMC12698881; doi:10.1007/s12264-025-01402-7)
Supplement: Supplementary file 1 — Supplementary file1 (PDF 401 KB) [file 12264_2025_1402_MOESM1_ESM.pdf]

Supplementary Materials

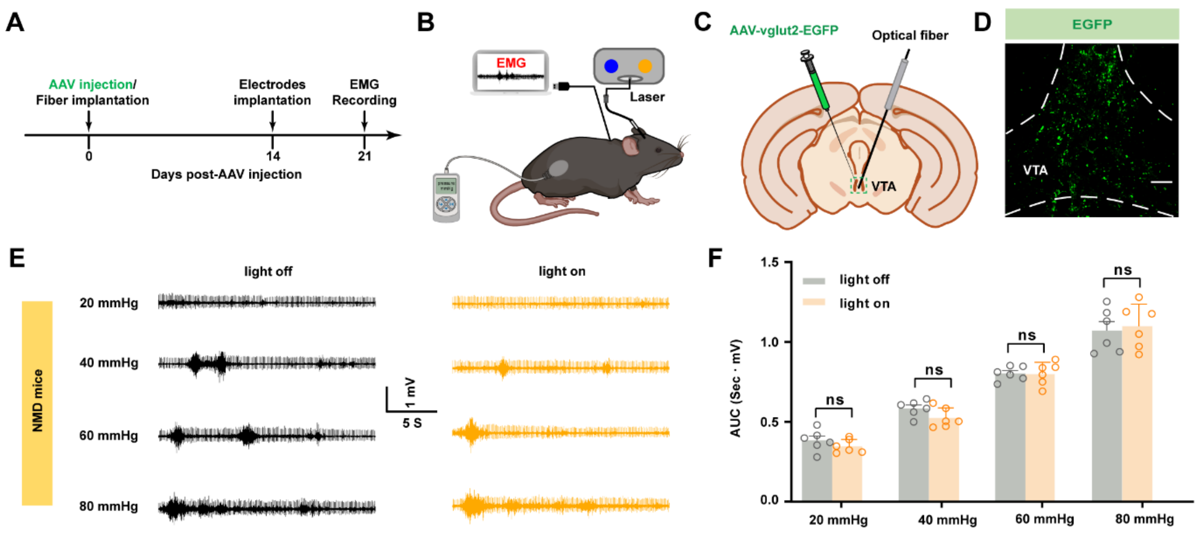

Supplementary Fig. S1. Optogenetic manipulation does not change visceral pain following VTA injection of the control virus in NMD mice. A Flowchart of optogenetic manipulation of glutamatergic VTA neurons in NMD mice. **B** Cartoon of EMG recording for assessing visceral pain. **C** Schematic of virus injection and optical fiber implantation at the VTA. **D** Representative image of virus expression in glutamatergic VTA neurons (scale bar, 100 μm). **E** Representative EMG traces from NMD mice at different levels of CRD stimulation (20, 40, 60, and 80 mmHg). **F** The area under the curve of the EMG in NMD mice at 20, 40, 60, and 80 mmHg (P >0.05, two-way ANOVA followed by Sidak's multiple comparisons test, n = 6 per group). ns, no significant difference, P >0.05.

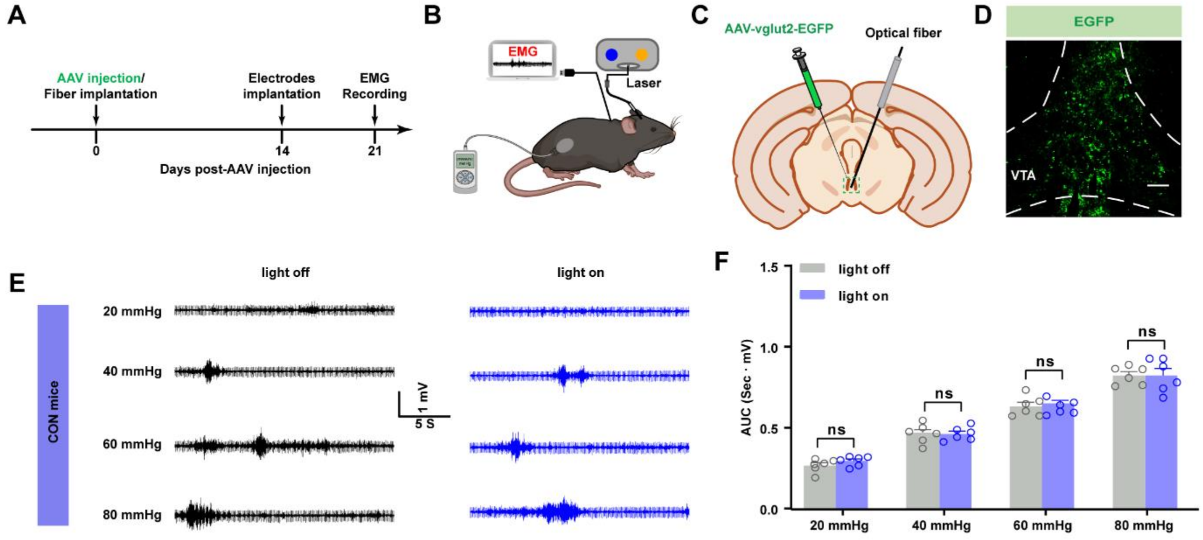

Supplementary Fig. S2. Optogenetic manipulation does not alter visceral pain following VTA injection of a control virus in CON mice. A Flowchart of optogenetic manipulation of glutamatergic VTA neurons in CON mice. **B** EMG recording for assessing visceral pain. **C** Schematic of virus injection and optical fiber implantation in the VTA. **D** Representative image of virus expression in glutamatergic VTA neurons (scale bar, 100 μm). **E** Representative EMG traces of CON mice at different levels of CRD stimulation (20, 40, 60 and 80 mmHg). **F** The area under the curve of the EMG in CON mice at 20, 40, 60, and 80 mmHg (*P* >0.05, two-way ANOVA followed by Sidak's multiple comparisons test, *n* = 6 per group). ns, no significant difference, *P* >0.05.
